# TAS-116 inhibits oncogenic KIT signalling on the Golgi in both imatinib-naïve and imatinib-resistant gastrointestinal stromal tumours

**DOI:** 10.1038/s41416-019-0688-y

**Published:** 2019-12-20

**Authors:** Yurina Saito, Tsuyoshi Takahashi, Yuuki Obata, Toshirou Nishida, Shuichi Ohkubo, Fumio Nakagawa, Satoshi Serada, Minoru Fujimoto, Tomoharu Ohkawara, Takahiko Nishigaki, Takahito Sugase, Masahiro Koh, Tomo Ishida, Koji Tanaka, Yasuhiro Miyazaki, Tomoki Makino, Yukinori Kurokawa, Kiyokazu Nakajima, Makoto Yamasaki, Seiichi Hirota, Tetsuji Naka, Masaki Mori, Yuichiro Doki

**Affiliations:** 1 Department of Gastroenterological Surgery, Osaka University Graduate School of Medicine, Suita, Japan; 2 National Cancer Center Hospital, Tsukiji, Japan; 3 Taiho Pharmaceutical Co. Ltd, Tsukuba, Japan; 4 Kochi Medical School Hospital, Nankoku, Japan; 5 Department of Surgical Pathology, Hyogo College of Medicine, Nishinomiya, Japan

**Keywords:** Sarcoma, Chaperones

## Abstract

**Background:**

Despite the effectiveness of imatinib mesylate (IM), most gastrointestinal stromal tumours (GISTs) develop IM resistance, mainly due to the additional kinase-domain mutations accompanied by concomitant reactivation of KIT tyrosine kinase. Heat-shock protein 90 (HSP90) is one of the chaperone molecules required for appropriate folding of proteins such as KIT.

**Methods:**

We used a novel HSP90 inhibitor, TAS-116, which showed specific binding to HSP90α/β with low toxicity in animal models. The efficacy and mechanism of TAS-116 against IM-resistant GIST were evaluated by using IM-naïve and IM-resistant GIST cell lines. We also evaluated the effects of TAS-116 on the other HSP90 client protein, EGFR, by using lung cell lines.

**Results:**

TAS-116 inhibited growth and induced apoptosis in both IM-naïve and IM-resistant GIST cell lines with KIT activation. We found KIT was activated mainly in intracellular compartments, such as *trans*-Golgi cisternae, and TAS-116 reduced autophosphorylated KIT in the Golgi apparatus. In IM-resistant GISTs in xenograft mouse models, TAS-116 caused tumour growth inhibition. We found that TAS-116 decreased phosphorylated EGFR levels and inhibited the growth of EGFR-mutated lung cancer cell lines.

**Conclusion:**

TAS-116 may be a novel promising drug to overcome tyrosine kinase inhibitor-resistance in both GIST and EGFR-mutated lung cancer.

## Background

Gastrointestinal stromal tumours (GISTs) are the most common gastrointestinal mesenchymal tumours.^1^ Approximately 85% of advanced GISTs have activating mutations in *KIT*, whereas another 5% harbour mutations in platelet-derived growth factor receptor α (*PDGFRA*).^2^ Imatinib mesylate (IM), a selective tyrosine kinase inhibitor (TKI), has been the standard therapy for patients with advanced, inoperable GISTs, and it yields long-lasting responses in the majority of patients, with a median survival of almost 5 years.^3,4^ However, 20% of patients show primary resistance to IM, and half of the responding patients eventually develop secondary resistance and show progression for about 2 years.^5–7^ GIST patients resistant to IM are basically treated with another TKI, sunitinib malate or regorafenib, which have been reported to prolong progression-free survival for 27.3 weeks and 4.8 months, respectively.^8,9^ Eventually, most patients develop resistance to these drugs. Such patients can develop progressing lesions at multiple metastatic sites, and each of the metastases in a patient can have different genomic mutational resistance mechanisms.^10–12^ Furthermore, sensitivity to TKIs varies considerably depending on the type of secondary mutation.^7,13^ One of the mechanisms of acquired resistance to EGFR-targeted inhibitors in EGFR-mutated lung cancer is the occurrence of a secondary mutation (T790M, C797S) in the kinase domain.^14^ Therefore, it is essential to validate novel therapeutic strategies.

Heat-shock proteins help the nascent polypeptide chain attain a functional conformation and facilitate protein stability, trafficking and the proteolytic turnover necessary for protein intracellular localisation and function.^15^ Heat-shock protein 90 (HSP90) regulates the conformation, function and activation of several client proteins including KIT and EGFR.^16,17^ Many of these functions are dysregulated in cancer; therefore, targeting HSP90 may be a novel strategy to modulate the growth of certain malignancies.^17^ HSP90 activity is regulated by many cofactors (co-chaperones) as well as conformational changes requiring ATP hydrolysis via the ATPase function of HSP90.^18^ HSP90 inhibitor molecules act by competitively blocking HSP90 enzymatic activity, resulting in the degradation of HSP90 client proteins.^16,19,20^ HSP90 inhibitors have been reported to have anti-tumour activity in breast and prostate cancer, multiple myeloma and melanoma models.^21–24^ Several HSP90 inhibitors in different malignancies are currently being studied.^25^ Because KIT and PDGFRA are clients for HSP90, HSP90 inhibitors induce KIT degradation and are expected to be a novel strategy for GIST treatment.^26–28^ In diverse GIST cell lines, HSP90 inhibition has been proven to efficiently kill tumour cells.^29–31^

The first-generation HSP90 inhibitor, 17-AAG, can cause severe hepatotoxicity.^32^ Neurological toxicities such as syncope and dizziness have been observed upon using the second-generation HSP90 inhibitor BIIB021.^33^ In clinical trials of other HSP90 inhibitors, the most commonly observed adverse events have been reversible visual disorders, which interfere with obtaining sufficient drug exposure, because of drug interruption or discontinuation or dose reduction.^32–36^

TAS-116 is an orally active ATP-competitive inhibitor of HSP90α and β.^37^ It shows greater specific binding to HSP90α and β than to highly homologous HSP90 family members GRP94 and TRTP1. TAS-116 shows anti-tumour activity in multiple xenograft models;^37^ however, anti-tumour activity against GIST is still unclear. In addition, it shows anti-tumour activity without detectable ocular toxicities in rats unlike other HSP90 inhibitors.^37^ These data suggest that TAS-116 has potential for clinical use; however, the mechanism underlying TAS-116 activity is still unclear. Therefore, the aim of this study was to clarify the effectiveness and the mechanism of TAS-116 activity in both IM-naïve and IM-resistant GISTs. Furthermore, to explore the effects of TAS-116 on other cancers, we evaluated its effects on EGFR-mutated lung cancer.

## Methods

### Cell culture

We used the established human GIST cell line GIST-T1 (Cosmobio, Tokyo, Japan), the identity of which was confirmed by DNA fingerprinting using short tandem repeat profiling.^38^ This *KIT* exon 11 mutant cell line is characterised by a heterozygous deletion of 57 bases.^39^ GIST-T1 cells were cultured in Dulbecco's modified Eagle's medium supplemented with 10% foetal bovine serum (FBS) (HyClone Laboratories, Logan, UT) and 100 U/ml penicillin and 100 μg/ml streptomycin (Nacalai Tesque, Kyoto, Japan) at 37 °C in a humid atmosphere of 5% CO_2_. To generate IM-resistant cell lines, GIST-T1 cells were cultured with increasing concentrations of IM. IM-resistant cell lines, R2, R8 and R9,^40^ were established and maintained under a constant concentration of 1 μM IM. GIST48/820 cell line and GIST430/654 were kindly provided by Dr. J. Fletcher (Dana-Farber Cancer Institute, Boston, MA, USA). GIST48/820 is characterised by a *KIT* exon 11 (homozygous V560D) and exon 17 (heterozygous D820A) mutation. GIST430/654 is characterised by a KIT exon 11 (near-homozygous frame deletion) and exon 13 (heterozygous V654A). GIST48/820 cells were cultured in DMEM medium supplemented with 15% FBS, 100 U/ml penicillin, 1% glutamine (Gibco, Thermo Fisher Scientific, Waltham, MA, USA), 100 μg/ml streptomycin at 37 °C in a humid atmosphere of 5% CO_2_. GIST430/654 cells were cultured in DMEM medium supplemented with 15% FBS, 100 U/ml penicillin, 1% glutamine, 100 μg/ml streptomycin and 100 nM imatinib at 37 °C in a humid atmosphere of 5% CO_2_. NCI-H1975 cells and HCC827 cells were purchased from American Type Culture Collection (Manassas, VA). A549 were purchased form Dainippon Pharmaceutical Co. Ltd. (Osaka, Japan). Those cells were cultured in RPMI 1640 medium supplemented with 10% FBS and 100 U/ml penicillin and 100 μg/ml streptomycin at 37 °C in a humid atmosphere of 5% CO_2_.

### Cell proliferation assay

Cells were plated in 96-well plates at a destiny of 3 × 10^3^ cells per well and incubated for 24 h. Cell proliferation was evaluated with WST-8 [2-(2-methoxy-4-nitro-phenyl)-3-(4-nitrophenyl)-5-(2,4-disulfophenyl)-2 H-tetrazolium, monosodium salt] assays (Cell Counting Kit-SF; Nacalai Tesque) at the indicated time points after treatment. The absorption of WST-8 was measured at a wavelength of 450 nm with a reference wavelength of 630 nm by using a microplate reader (Model 680; Bio-Rad Laboratories). The growth rate was expressed as the percentage of absorbance for treated cells versus that of control cells. Experiments were performed with six replicate wells for each sample, and the data are presented as averages.

### Western blotting analysis

Cell lines and tumour tissues harvested from the xenograft mouse model were lysed in RIPA buffer (10 mM Tris-HCl [pH 7.5], 150 mM NaCl, 1% Nonidet P-40, 0.1% sodium deoxycholate, 0.1% sodium dodecyl sulfate (SDS), 1 × phosphatase inhibitor cocktail [Nacalai Tesque] and 1 × protease inhibitor cocktail [Nacalai Tesque]) followed by centrifugation at 14,000 × *g* for 15 min at 4 °C. The supernatants were stored at −80 °C until use. Protein concentrations were determined with a DC Protein Assay kit (Bio-Rad Laboratories, Hercules, CA) using bovine serum albumin (BSA) as the concentration standard. Proteins were resolved using SDS-polyacrylamide gel electrophoresis (SDS-PAGE) with gels purchased from Wako Pure Industries (Osaka, Japan) or Bio-Rad Laboratories (Hercules, CA) and subsequently transferred to polyvinylidene difluoride membranes (Millipore, Bedford, MA). The membranes were blocked with 5% skim milk in Tris-buffered saline containing 0.1% Tween 20 and incubated with the respective antibodies against different targets. The following antibodies were used: anti-phospho-c-KIT (Y703), anti-phospho-AKT (S473), anti-AKT, anti-phospho-p44/42 mitogen-activated protein kinase (MAPK) (T202/Y204), anti-p44/42 MAPK, anti-phospho-EGFR (Y1068), anti-EGFR (all from Cell Signaling Technology, Danvers, MA), anti-cKIT (C-19), anti-HSP90, anti-GAPDH (all from Santa Curz Biotechnology, Dallas, TX) and anti-HSP70 antibodies (from StressGen Biotechnologies Corporation, Victoria, BC). Next, the membranes were incubated with horseradish peroxidase-conjugated sheep anti-mouse IgG, horseradish peroxidase-conjugated (HRP-conjugated) donkey anti-rabbit IgG (GE Healthcare, Little Chalfont, Buckinghamshire, UK) or HRP-conjugated goat anti-rabbit IgG (Cell Signaling Technology, Danvers, MA). Finally, the signals were visualised using an enhanced chemiluminescence reaction system (Perkin-Elmer Life Sciences, Boston, MA).

### Caspase-3/7 activity assay

GIST cell lines were plated into 96-well white plates at a density of 3 × 10^3^ cells per well and treated with IM and TAS-116 for 24 h. The activities of caspase-3 and caspase-7 in cell culture were detected using Caspase Glo 3/7 Assays (Promega, Madison, WI) according to the manufacturer’s instructions. Luminometer readings were determined 1 h after addition of the reagent by using a Spectra Max Gemini EM Microplate Reader (Molecular Devices, Sunnyvale, CA).

### Immunofluorescence confocal microscopy

The following antibodies were purchased: anti-KIT (M-14) from Santa Cruz Biotechnology (Dallas, TX); anti-KIT (D13A2) and anti-phospho-KIT (Y703) from Cell Signaling Technology (Danvers, MA); anti-GM130^34^ from BD Transduction Laboratories (Franklin Lakes, NJ) and anti-PDI from Abcam (Cambridge, UK). Alexa Fluor-conjugated secondary antibodies were obtained from Molecular Probes (Eugene, OR).

Cells cultured on poly-L-lysine-coated coverslips were fixed with 4% paraformaldehyde for 20 min at 25 °C. The fixed cells were permeabilised and blocked for 30 min in PBS supplemented with 0.1% saponin and 3% BSA, and then incubated with a primary and secondary antibody for 1 h each. Confocal images were obtained using a Fluoview FV10i laser scanning microscope with an x60 1.20 N.A. water-immersion objective (Olympus, Tokyo, Japan). Composite figures were prepared with Photoshop Elements 10 and Illustrator CS6 software (Adobe, San Jose, CA).

### Analysis of protein glycosylation

Following the manufacturer’s instructions (New England Biolabs, Ipswich, MA), NP-40 cell lysates were treated with endoglycosidases for 1 h at 37 °C. The reactions were stopped with SDS-PAGE sample buffer; the products were resolved by SDS-PAGE and immunoblotted with the anti-KIT antibody.

### GIST cell xenograft mouse models

In this study, since it is essential to provide the efficacy and safety of this drug in animal model, we carried out animal experiments. All animal experiments were conducted according to the institutional ethical guidelines for animal experimentation of Taiho Pharmaceutical Co., Ltd. Mice were housed in a temperature-controlled room with 12 h light/12 h dark cycle and provided free access to water and diets in the laboratory of Taiho Pharmaceutical Co., Ltd. GIST T1 and R8 cells were implanted in BALB/cAJcl-nu/nu mice obtained from CLEA Japan Inc. (Tokyo, Japan), and GIST R9 cells were implanted in C.B-17/lcr-scid/scidJcl mice obtained from CLEA Japan Inc. (Tokyo, Japan). For cell inoculation, 1.0 × 10^7^ cells were injected subcutaneously into the flanks of the mice. When the tumour volume reached approximately 150 mm^3^, the mice were randomly divided into three groups. The animals were treated orally for 3 weeks with control (0.5 w/v% hydroxypropyl methyl cellulose every day), IM (50 mg/kg twice daily every day) and TAS-116 (14 mg/kg once daily five times a week) at home cage. Drug dose used was determined according to the previous report.^37^ Tumour sizes were measured twice a week, and body weight was measured at the same time throughout the study. Tumour volumes were determined by measuring the length (*L*) and width (*W*) and calculated as (*W*^2^ × *L*)/2. Tumours were resected 22 days after the first treatment. For the xenograft experiments, the mice were anaesthetised by 3% isoflurane in invasive procedure and killed by standard CO_2_ asphyxiation.

### Immunohistochemistry

Subcutaneously implanted tumours were harvested and embedded in paraffin for immunohistochemical analysis using the CD117 polyclonal rabbit immunostain (Dako Corporation, Carpinteria, CA) and anti-Ki67 antibodies (Novocastra Laboratories, Newcastle, UK). Terminal dUTP nick-end labelling (TUNEL) assays (with DAPI Fluorescein In Situ Apoptosis Detection Kit [Chemicon International, Temecula, CA] were performed according to the manufacturer’s instructions.

### Statistical analysis

Data are shown as mean ± SD values for in vitro and in vivo experiments. To test for statistically significant differences between the two groups, the Tukey–Kramer honestly significant difference test was used. Differences were considered significant at *P* < 0.05. All analyses were performed using JMP version 11.0 (SAS Institute, Cary, NC).

## Results

### Inhibition of GIST cell proliferation by TAS-116 treatment

**Fig. 1:**
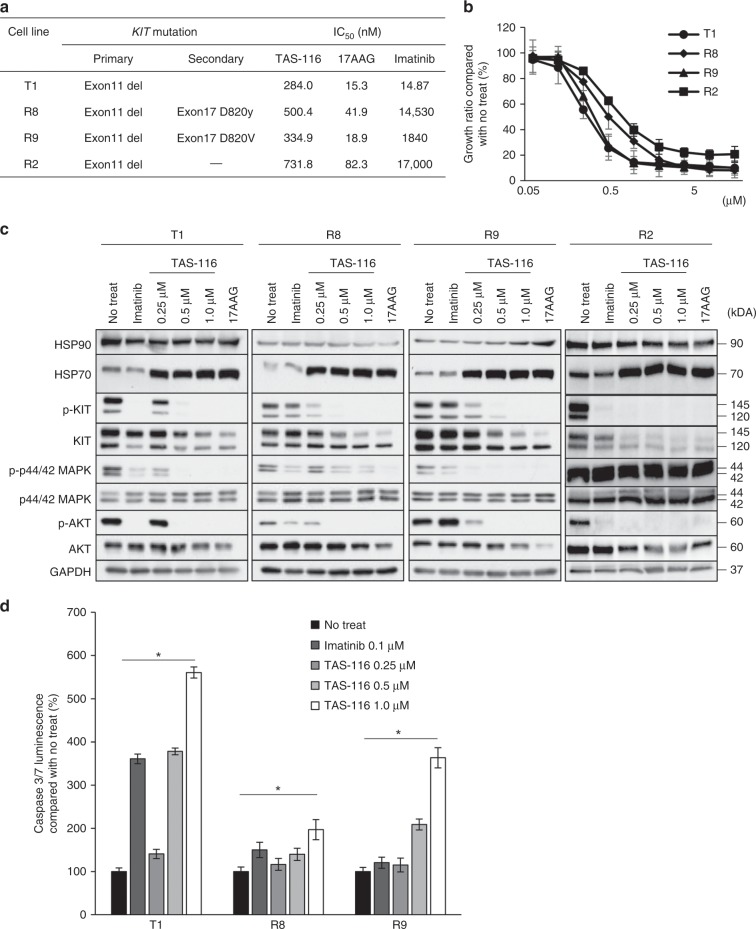
Effect of HSP90 inhibition on mutant KIT signalling. **a** *KIT* gene mutation and effects of IM, TAS-116 and 17-AAG in IM-naïve and IM-resistant GIST cell lines. **b** Cell proliferation was determined by WST-8 assays at 72 h after incubation with TAS-116. Each value is represented as the mean ± SD. **c** Western blot analysis of HSP90, HSP70, pKIT/KIT, pAKT/AKT and pERK/ERK in GIST cell lysates at 12 h after IM, TAS-116 and 17-AAG treatments was performed. **d** Caspase-3/7 activity was determined using luminescence assays 24 h after incubation with 0.1 μM IM and 0.25–1.0 μM TAS-116.

To analyse the growth inhibitory mechanism of TAS-116 in GIST cells, we used IM-naïve GIST T1 and IM-resistant GIST R8, R9, and R2. GIST R8 and R9 cells have KIT secondary mutation D820Y and D820V, respectively, whereas GIST R2 cells do not have KIT secondary mutation. The IC_50_ values of TAS-116 for GIST T1, R8, R9 and R2 were 284.0, 500.4, 334.9 and 731.8 nM, respectively (Fig. 1a). Figure 1b shows that TAS-116 inhibited the proliferation of IM-naïve and IM-resistant GIST cell lines similarly in a dose-dependent manner, whereas it inhibited the proliferation of GIST R2 cells to a lesser extent, in which the growth is not dependent on KIT activation under imatinib existence (Fig. 1c). The immunoblotting analysis showed that TAS-116 treatment induced HSP70 expression, an indicator of HSP90 inhibition, in GIST T1, R8, R9 and R2 cells. IM significantly inhibited KIT phosphorylation and the subsequent signalling pathways in the IM-naïve cell line (T1). In contrast, TAS-116 decreased total KIT, as well as phosphorylated KIT (pKIT) levels and the downstream signalling pathways, such as phosphorylated p44/42 MAPK and AKT (T1, R8 and R9). Similar findings were obtained in GIST48/820 and GIST430/654 (Supplementary Fig. 1a, b). Importantly, pKIT appeared to be more sensitive to TAS-116 and was more prominently decreased than total KIT. In contrast, although TAS-116 decreased total KIT as well as phosphorylated KIT (pKIT) levels in R2 cells, the downstream signalling pathways, such as phosphorylated p44/42 MAPK, were not changed (Fig. 1c).

To evaluate the induction of apoptosis by TAS-116, we measured the activity of caspase-3/7 by using luminescence assays. Caspase 3/7 activity increased in a dose-dependent manner after exposure to TAS-116 and was higher in GIST T1 than in GIST R8 and R9 cell lines (Fig. 1d).

### Effects of HSP90 inhibitors on cellular distribution of KIT

**Fig. 2:**
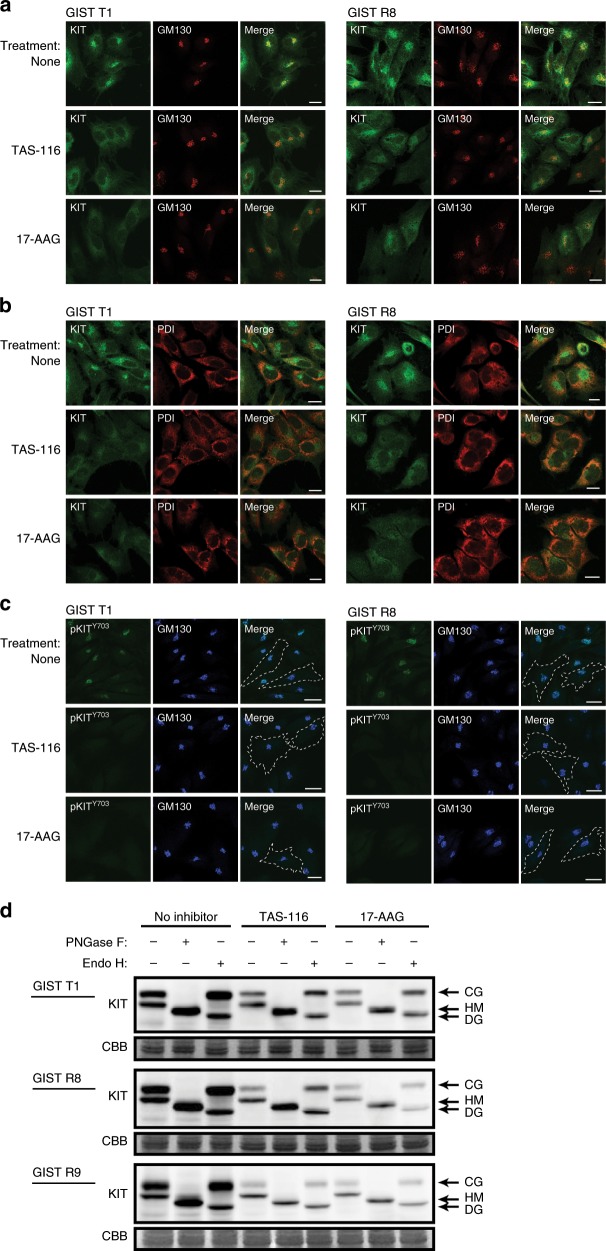
Effect of HSP90 inhibition on KIT distribution and phosphorylated KIT in GIST T1 and GIST R8 cells. **a**–**d** GIST cells were treated with 1 μM TAS-116 or 0.5 μM 17-AAG for 12 h. **a**–**c** The cells were stained with anti-KIT (green), anti-phosphorylated KIT Y^703^ (anti-KIT Y^703^, green), anti-GM130 (Golgi marker, red or blue) and anti-PDI antibodies (ER marker, red). Dashed lines indicate cell borders. Bars, 20 μm. **d** Lysates from GIST cell lines were treated with peptide N-glycosidase F (PNGase F) or endoglycosidase H (Endo H) and then immunoblotted with anti-KIT antibody. CG complex-glycosylated form, HM high-mannose form, DG deglycosylated form.

We reported that mutated KIT in GIST cell lines accumulated on the *trans*-side of the Golgi apparatus^41^ where mutated KIT was phosphorylated on Y703 predominantly, but not on the endoplasmic reticulum (ER).^42^ Next, we examined the effects of the HSP90 inhibitors on KIT localisation and phosphorylation using confocal microscopy. GM130 is Golgi marker and PDI is ER marker. Treatment with TAS-116 or 17-AAG decreased Golgi-localised KIT in both IM-naïve GIST T1 and IM-resistant GIST R8 cells, but KIT remained to be co-localised with PDI (ER) in HSP90-inhibited cells (Fig. 2a, b). KIT phosphorylated on Y703 almost completely disappeared from the Golgi apparatus following the addition of HSP90 inhibitors (Fig. 2c). Similar results were obtained using GIST48/820 and GIST430/654 (Supplementary Fig. 1c, d) and GIST R9 (Supplementary Fig. 2a–c).

We used peptide-N-glycosidase F (PNGase F), which completely digest N-linked glycans, to show that the lower and upper KIT bands contained different glycans. We also treated KIT with endoglycosidase H (endo H), which digests immature high-mannose forms, but not mature complex-glycosylated forms of KIT. We previously showed by in vitro glycosidase treatment and immunoblotting that the lower and upper KIT bands are high-mannose form (HM, endo H-sensitive, ER and *cis*-side of Golgi) and complex-glycosylated form (CG, endo H-resistant, reaching *trans*-side of Golgi), respectively.^41^ TAS-116 and 17-AAG decreased both CG-KIT and HM-KIT, but the decrease in the CG form was greater than that in the HM form (Fig. 2d). As KIT is fully activated on the *trans*-Golgi in the CG form, these HSP90 inhibitors predominantly decrease KIT on the *trans*-Golgi compared with that on the ER and *cis*-Golgi.

### Anti-tumour effects of TAS-116 in GIST xenograft models

**Fig. 3:**
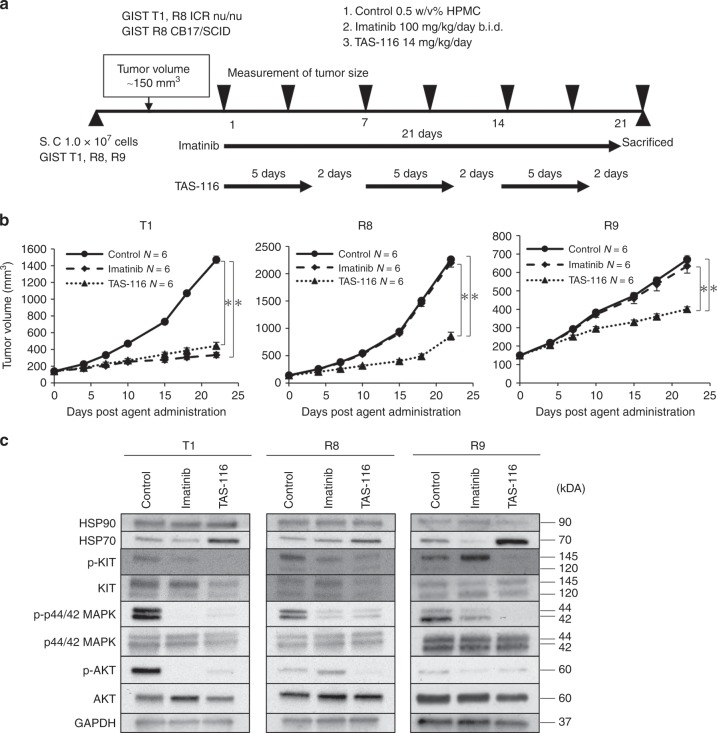
TAS-116 had anti-tumour effects in xenograft mouse models. **a** GIST cell line xenograft mouse model; BALB/cAJcl-nu/nu mice (6 weeks of age) were injected with 1.0 × 10^7^ cells of GIST T1 and GIST R8, whereas C.B-17/lcr-scid/scidJcl mice (7 weeks of age) were injected with 1.0 × 10^7^ cells of GIST R9. When the tumour volume reached ~150 mm^3^, xenograft tumours were treated with TAS-116 five times a week orally at 14 mg/kg and IM twice a day orally at 50 mg/kg for 3 weeks. **b** Tumour volumes were determined twice per week. Statistical analysis was performed using the Tukey-Kramer (HSD) test (*p < 0.01). **c** Western blot analysis of HSP90, HSP70, pKIT/KIT, pAKT/AKT and pERK/ERK in GIST cell-derived tissues from control, IM-treated and TAS-116–treated animals.

We evaluated the in vivo effects of TAS-116 against GIST tumours using a xenograft mouse model as shown in Fig. 3a. Both IM and TAS-116 significantly inhibited tumour growth in the GIST T1 xenograft model (*p* *<* 0.0001 and <0.0001, respectively), whereas TAS-116, but not IM, significantly suppressed tumour growth in the GIST R8 and R9 models (*p* *<* 0.0001 and <0.0001, respectively) (Fig. 3b). Weight loss after TAS-116 treatment was not observed compared with that before treatment in any of the xenograft models.

**Fig. 4:**
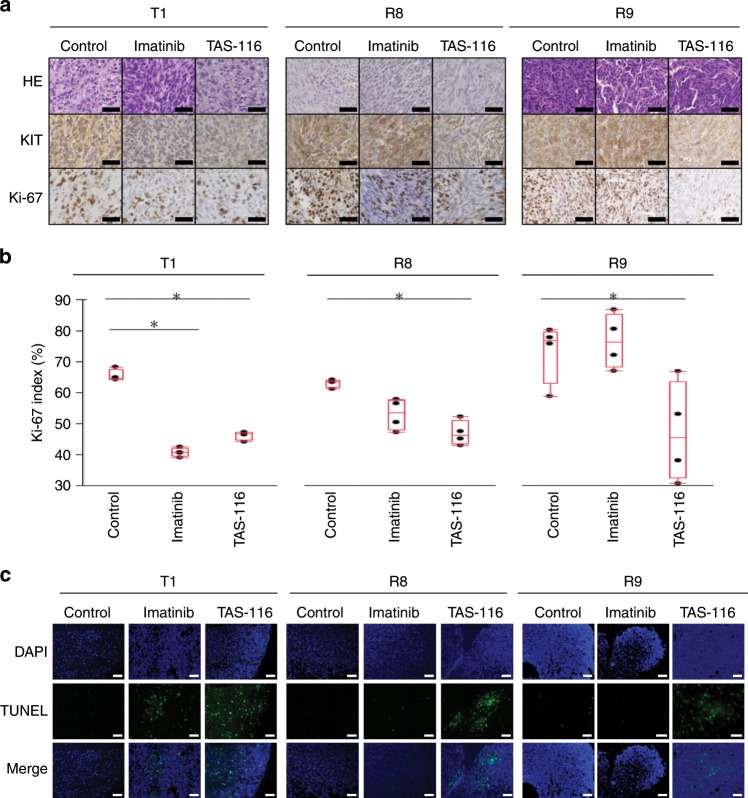
Histological analysis of IM and TAS-116 in GIST cell xenograft mouse models. **a** Immunohistochemical analysis of KIT and Ki-67 in GIST cell xenograft mouse-derived tissue from control, IM-treated and TAS-116–treated animals. **b** Ki-67 staining was recorded as the ratio of positively stained cells to all tumour cells in five fields (×200 magnification). Statistical analysis was performed using the Tukey-Kramer (HSD) *t*-test (*p < 0.01). **c** Analysis of apoptosis by TUNEL staining (blue fluorescence, DAPI staining for nuclei; green fluorescence, TUNEL-positive staining) in GIST cell xenograft mouse-derived tissue from control, IM-treated and TAS-116–treated animals. Scale bar = Black 50 μm, White 100 μm.

We next examined the expression and phosphorylation of KIT and its downstream signalling pathways in xenograft mouse models (Fig. 3c). Not only pKIT but also its downstream signalling pathways, including AKT and p44/42 signalling, were suppressed by TAS-116 treatment. The harvested subcutaneous tumours were histologically examined (Fig. 4a). Expression of KIT was attenuated in the IM-treated GIST T1 xenograft model and in TAS-116-treated GIST T1, R8, and R9 xenografts. More importantly, Ki-67, a proliferation marker, was significantly decreased in the IM-treated GIST T1 xenograft (*p* < 0.0001) and TAS-116-treated GIST T1, R8, and R9 xenografts (*p* < 0.0001, =0.0005, and =0.0326, respectively) (Fig. 4a, b). Consistent with the above results, TUNEL staining showed that cellular apoptosis was induced in the IM-treated GIST T1 xenograft and TAS-116-treated GIST T1, R8 and R9 xenografts (Fig. 4c).

### TAS-116 suppresses the proliferation of lung cancer cell lines with EGFR mutations

**Fig. 5:**
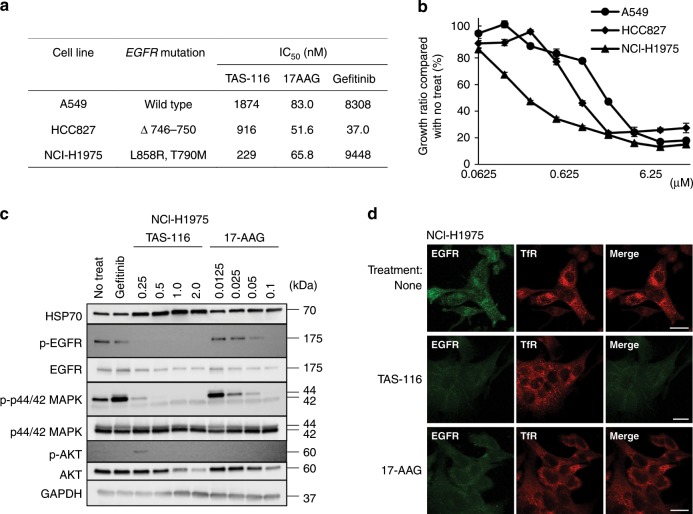
Effects of HSP90 inhibition on mutant EGFR signalling. **a** EGFR gene mutation and effects of gefitinib, TAS-116 and 17-AAG in gefitinib-naïve and gefitinib-resistant lung cancer cell lines. **b** Cell proliferation was determined by WST-8 assays at 72 h after incubation with TAS-116. Each value is represented as the mean ± SD. **c** Western blot analysis of HSP70, pEGFR/EGFR, pAKT/AKT and pERK/ERK in NCI-H1975 cell lysates at 24 h after gefitinib, TAS-116 or 17-AAG treatment was performed. **d** NCI-H1975 cells were treated with 2 μM TAS-116 or 0.1 μM 17-AAG for 24 h. Cells were stained with anti-EGFR (green), and anti-TfR antibodies (endosome marker, red). Bars, 20 μm.

To confirm the activities of TAS-116 on other tyrosine kinases, we evaluated growth inhibition by TAS-116 using several lung cancer cell lines, including EGFR-wild-type and EGFR-mutated cell lines. IC_50_ values of TAS-116, 17-AAG, and gefitinib were measured using the EGFR-wild-type cell line A549, gefitinib-naïve HCC827 (EGFR Δ746-750) and gefitinib-resistant NCI-H1975 (EGFR L858R/T790M) (Fig. 5a). As shown in Fig. 5b, TAS-116 inhibited the proliferation of all lung cancer cell lines in a dose-dependent manner. Among the three cell lines, NCI-H1975 (EGFR L858R/T790M) appeared to be the most sensitive to TAS-116, whereas it had mild effects on the EGFR-wild-type A549 cell line. Immunoblotting showed that TAS-116 significantly induced HSP70 in NCI-H1975 cells. TAS-116 inhibited phosphorylated EGFR and its downstream signalling in NCI-H1975 cells (Fig. 5c).

Next, we examined the localisation of EGFR after treatment with TAS-116. Before the treatment, EGFR in NCI-H1975 cells was predominantly co-localised with an endosome marker, transferrin receptor and treatment with TAS-116 or 17-AAG decreased EGFR expression in the endosomes (Fig. 5d).

## Discussion

Activation mutation of *KIT* or *PDGFRA* receptor tyrosine kinases is an initiating oncogenic event and it plays important roles in the proliferation of GISTs.^43^ This led to the development of several clinically used KIT-targeting small-molecule TKIs.^3,4,8,9^ HSP90 is a molecular chaperone that is crucial for many oncogenic drivers such as KIT and EGFR.^27^ HSP90 inhibitors, including IPI-493, IPI-504, AT13387, and AUY922, have shown inhibitory effects on GIST cell growth in preclinical studies.^30,31,44,45^ These inhibitors, however, have also been shown to have limited activity in clinical settings as they cause adverse events. TAS-116, an oral selective HSP90α/β inhibitor, showed significant anti-tumour activities without detectable ocular toxicities in a rat model.^37^ A Phase 1 trial of TAS-116 in patients with solid tumours demonstrated that TAS-116 had an acceptable safety and preliminary anti-tumour activity.^46^ The present study provides evidence that TAS-116 shows activity against GIST cell line GIST T1 and EGFR-mutated lung cancer cells.

Despite the efficacy of IM, half of the GISTs treated with IM show resistance within 2 years. This IM resistance is mainly caused by secondary mutations in the kinase domains of *KIT* and is accompanied by concomitant reactivation of KIT even in the presence of IM, suggesting that KIT oncoprotein remains a therapeutic target at the time of clinical progression.^10^ Mutated forms of the proteins are also dependent on HSP90 for their stability.^47^ Fumo et al. reported that HSP90 inhibition by 17-AAG destabilised KIT and led to rapid degradation of the IM-resistant *KIT* mutant protein with D816V mutation.^47^ Smyth et al. showed that AT13387 had significant anti-tumour activity in two IM-resistant cell lines, which have differently resistant *KIT* mutations (V654A and D820A).^30^ Thus, these studies indicate that HSP90 is a desirable target for GISTs with various secondary mutations in *KIT*. HSP90 inhibitors inhibited the proliferation of both IM-naïve and IM-resistant GIST, irrespective of the type of *KIT* mutation.^29^ We have established IM-resistant GIST cell lines with secondary KIT mutations at D820Y and D820V (GIST R8 and R9), which are mutations found in IM-resistant GISTs in clinical practice.^40^ In this study, we showed that TAS-116 exerted inhibitory activities on both IM-naïve and KIT-dependent IM-resistant GISTs, with a decrease in pKIT and its downstream signalling activities (e.g., AKT and ERK). In contrast, TAS-116 showed less activity in the KIT-independent IM-resistant GIST R2 cells. These results suggested that TAS-116 might inhibit the KIT-dependent proliferation of GISTs by the depletion of pKIT and that we may be able to predict the therapeutic effect of TAS-116 in IM-resistant GIST through the measurement of KIT activities in a clinical setting.

**Fig. 6:**
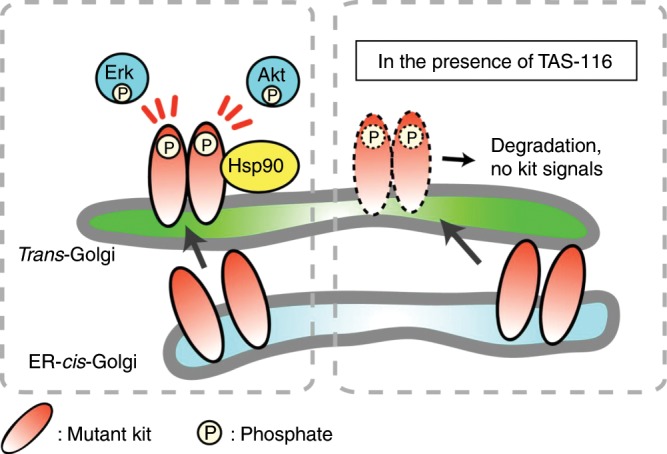
Model of mutant KIT signalling on intracellular compartments in GISTs. Newly synthesised mutant KIT traffics from the ER to the Golgi complex. After reaching the Golgi complex, mutant KIT can activate the PI3K-AKT pathway and ERK. Phosphorylated KIT in the Golgi complex requires HSP90 for stability. TAS-116 mediates the degradation of phosphorylated KIT.

We previously reported that mutant KIT accumulated on the Golgi apparatus, whereas normal KIT localised to the plasma membrane.^41^ Both IM-naïve and IM-resistant KIT became fully autophosphorylated on the Golgi apparatus in a complex-glycosylated form.^41^ In the present study, TAS-116 decreased fully glycosylated KIT on the *trans*-Golgi apparatus. There are four isoforms of HSP90 proteins and HSP90 inhibitors are reported to bind differently to these isoforms. TAS-116 was shown to have specific binding to cytosolic HSP90α/β.^37^ We postulated that this specificity of TAS-116 might correlate with the inhibitory activities of mutant KIT. In fact, pKIT, which was located in the *trans*-Golgi cisternae predominantly, decreased after TAS-116 treatment (Fig. 6).

We also showed that TAS-116 was effective against EGFR-mutated lung cancer by decreasing EGFR in endosomes. TAS-116 may be effective against tumours resistant to TKIs due to secondary mutations, such as in GISTs or EGFR-mutated lung cancer. In clinical trials, HSP90 inhibitors were reported to be effective against cancers with driver mutation products that are clients of HSP90, such as KIT, EGFR, ALK, HER2 and BRAF.^15^ Taken together, TAS-116 may be potentially active against TKI-resistant cancers with driver mutations, of which products are clients of HSP90α/β.

There are several limitations to this study. First, we examined only three GIST cell lines and two EGFR-mutated lung cancer cell lines. However, GIST cell lines are rare; especially IM-resistant GIST cell lines with secondary mutations established are quite unique. Second, TAS-116 decreased KIT protein levels on the Golgi apparatus; however, the effects of TAS-116 on the protein structure and mechanism of KIT degradation are still unknown; further experiments to elucidate how KIT protein is degraded on the Golgi apparatus by HSP90 inhibition are required.

Finally, we showed that TAS-116 has significant anti-tumour effects against both IM-naïve and IM-resistant xenograft models and is safe. The highly selective HSP90α/β inhibitor TAS-116 did not inhibit the interaction between GRP94 and LRP6, which may have resulted in decreased GRP94-related gastrointestinal toxicity.^37^ A Phase 2 clinical study has shown that TAS-116 is well-tolerated and has significant clinical benefits in patients with metastatic or unresectable GISTs refractory to standard therapies as a ≥4th-line treatment.^48^ Furthermore, a confirmatory trial is ongoing for its pharmaceutical approval.

In conclusion, our results showed that TAS-116 effectively inhibited the proliferation of IM-naïve and IM-resistant GIST cells and reduced the growth of GIST tumours in a xenograft mouse model. It was also effective against gefitinib-naïve and gefitinib-resistant EGFR-mutated lung cancer. These anti-tumour effects appear to be mediated by the downregulation of pKIT in the Golgi apparatus or EGFR in the endosomes. We propose that TAS-116 may be a promising drug to overcome TKI-resistant GISTs and lung cancer.

## Supplementary information

Supplementary data

## Data Availability

All data generated or analysed during this study are included in this published article and its supplementary information files.
