# Supplementary material for: TAS-116 inhibits oncogenic KIT signalling on the Golgi in both imatinib-naïve and imatinib-resistant gastrointestinal stromal tumours
Source: Br J Cancer. 2019 Dec 20;122(5):658–67. doi: 10.1038/s41416-019-0688-y (PMC7054534; doi:10.1038/s41416-019-0688-y)

Supplementary Figure 1. Effect of HSP90 inhibition on mutant KIT signaling. (*a*) *KIT* gene mutation and effects of IM, TAS-116, and 17-AAG in IM-resistant GIST cell lines. (*b*) Western blot analysis of HSP90, HSP70, pKIT/KIT, pAKT/AKT, and pERK/ERK in GIST cell lysates at 12 h after IM, TAS-116, and 17-AAG treatments was performed. (*c*-*d*) GIST cells were treated with 1 μM TAS-116 or 0.5 μM 17-AAG for 12 h. The cells were stained with anti-KIT (green), anti-phosphorylated KIT Y^{703} (anti-KIT Y^{703}, green), and anti-GM130 (Golgi marker, red or blue). Dashed lines indicate cell borders. Bars, 20 μm.

Supplementary Figure 2. Effects of HSP90 inhibition on KIT distribution in GIST R9 cells. (*a*-*c*) GIST R9 cells were treated with 1 μM TAS-116 or 0.5 μM 17-AAG for 12 h. Cells were stained with anti-KIT (green), anti-phosphorylated KIT Y703 (anti-KIT Y^{703}, green), anti-GM130 (Golgi marker, red or blue), and anti-PDI antibodies (ER marker, red). Dashed lines indicate cell borders. Bars, 20 μm.

Supplementary Figure 1

a

Cell line	*KIT* mutation		IC$_{50}$ (nM)		
	Primary	Secondary	TAS-116	17AAG	imatinib
GIST48/820	Exon11 V560D	Exon17 D820A	288.8	47.4	＞1000
GIST430/654	Exon11 del	Exon13 V654A	164.4	28.5	＞1000

b

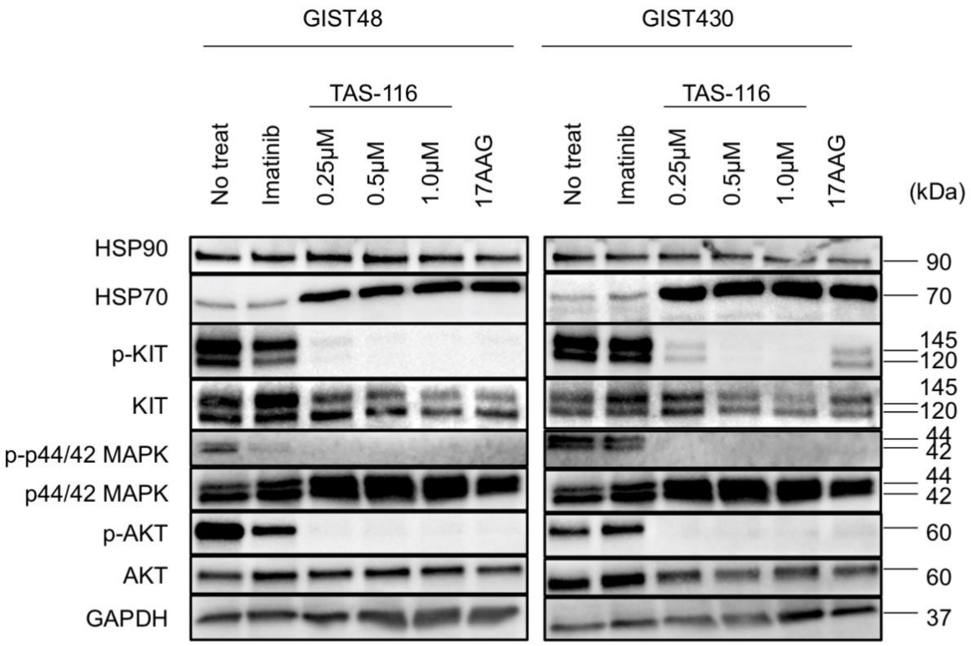

c

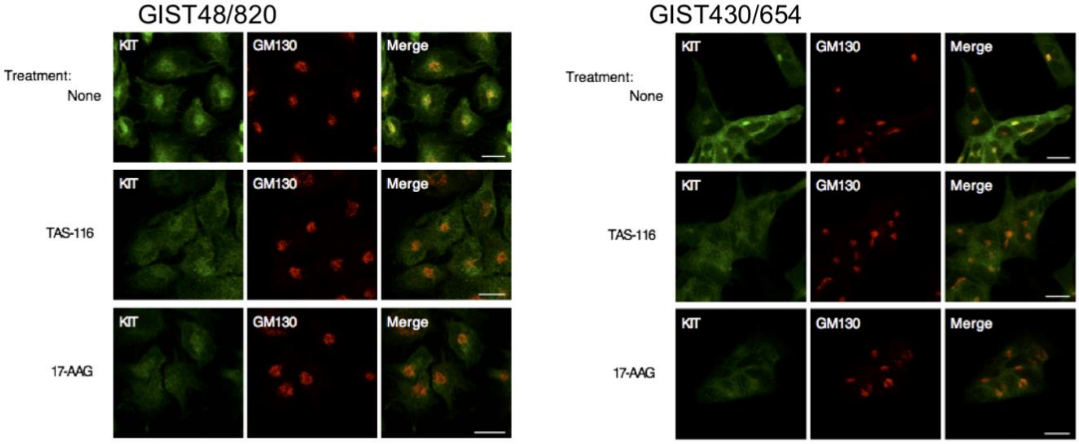

d

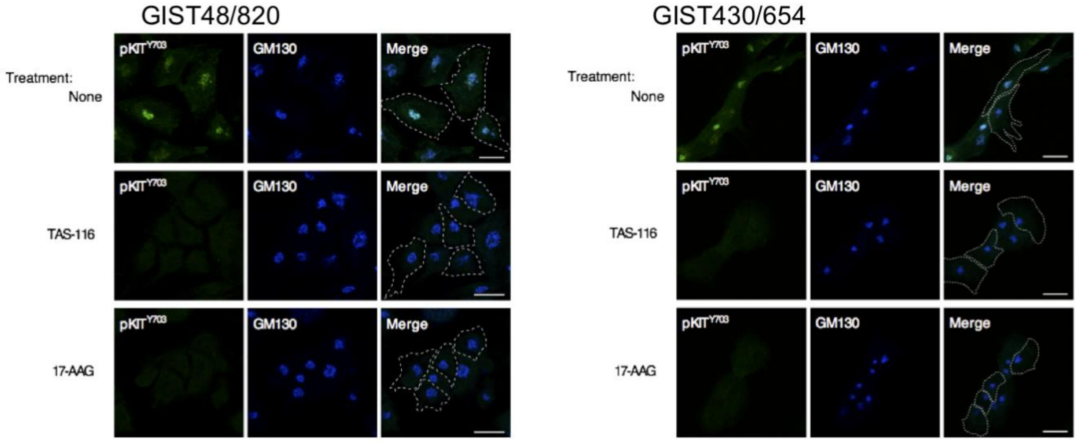

Supplementary Figure 2

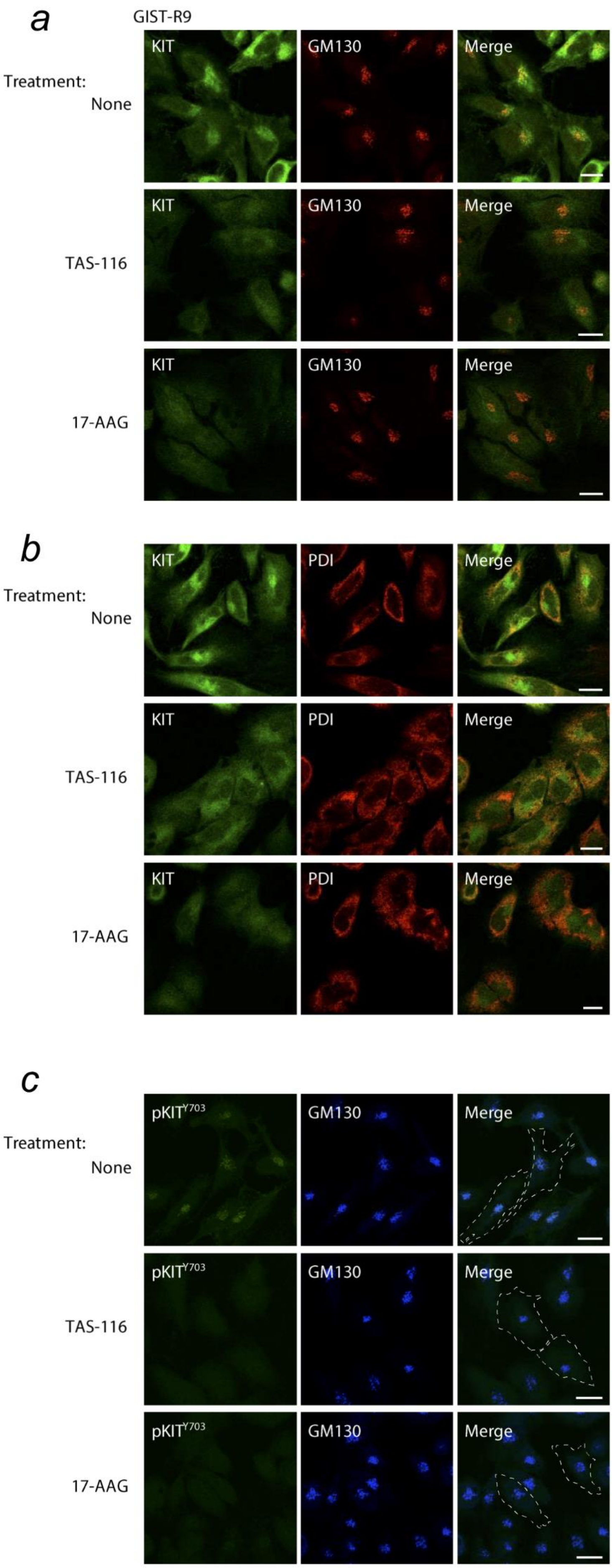

Supplement: Supplementary file 1 — Supplementary data [file 41416_2019_688_MOESM1_ESM.pdf]
